# Theoretical model of mitotic spindle microtubule growth for FRAP curve interpretation

**DOI:** 10.1186/s12918-016-0378-9

**Published:** 2017-02-24

**Authors:** Leonid V. Omelyanchuk, Alina F. Munzarova

**Affiliations:** 1 Institute of Molecular and Cellular Biology, Novosibirsk, Russia; 2 Novosibirsk State University, Novosibirsk, Russia

**Keywords:** Mitotic spindle, FRAP (fluorescence recovery after photobleaching), Microtubules, Fluorescently marked tubulin, Growing/shrinking microtubule ends, PDE (partial differential equation)

## Abstract

**Background:**

Spindle FRAP curves depend on the kinetic parameters of microtubule polymerization and depolymerization. The empirical FRAP curve proposed earlier permits determination of only one such dynamic parameter, commonly called the "tubulin turnover". The aim of our study was to build a FRAP curve based on an already known kinetic model of microtubule growth.

**Results:**

A numerical expression that describes the distribution of polymerizing and depolymerizing microtubule ends as a function of four kinetic parameters is presented. In addition, a theoretical FRAP curve for the metaphase spindle is constructed using previously published dynamic parameters.

**Conclusion:**

The numerical expression we elaborated can replace the empirical FRAP curve described earlier for a spindle comprising fluorescently marked microtubules. The curve we generated fits well the experimental data.

## Background

Fluorescence recovery after photobleaching (FRAP) was first introduced in 1974 [1] and is a widely used method to study turnover, transport, diffusion and interaction among biological molecules in living specimens. The use of FRAP has been facilitated by the current availability of microscopes equipped with a laser scanning device. The emergence of fluorescent protein labeling with the green fluorescent protein (GFP) and its spectral variants has greatly enhanced FRAP application. In a typical FRAP experiment, a GFP-labeled structure is rapidly and irreversibly photobleached with a high intensity laser, and fluorescence recovery is recorded as a function of time. The fluorescent molecules then diffuse into the irradiated region, while the non-fluorescent ones diffuse into the unbleached area until equilibrium is reached. The analysis of fluorescence recovery curves yields the diffusion coefficient and the fraction of free, transiently bound and immobilized molecules.

For a quantitative description of fluorescence recovery dynamics in FRAP experiments, several theoretical models have been proposed [2]. These include (1) the Pure-Diffusion Dominant Model that considers the recovery rate for weakly bound or free fluorescent molecules, and is defined exclusively by their diffusion; (2) the Effective Diffusion Model, which describes the recovery kinetics of fluorescent molecules that bind tightly the bleached structure, and is also largely defined by diffusion; (3) the Reaction Dominant Model, where diffusion is very fast and molecules rapidly equilibrate after the bleach; and (4) the Diffusion Phase-binding Phase approximation used whenever the contributions of diffusion and binding are coupled. In another early study, *Salmon* et al. [3] used the FRAP technique to trace the behavior of fluorescently labeled bovine tubulin injected in sea urchin eggs undergoing the first mitotic division. The authors provided evidence that the use of exogenous tubulin accurately represents the in vivo situation, and showed that FRAP data were best fitted by a negative-going exponential function, and that fluorescence recovery had a half-time of approximately 20 s. Taking into account that tubulin dimers diffused back into the bleached area within 1 s, the authors concluded that diffusion was not a limiting factor and that the Reaction Dominant Model was correctly interpreting their FRAP results.

**Fig. 1:**
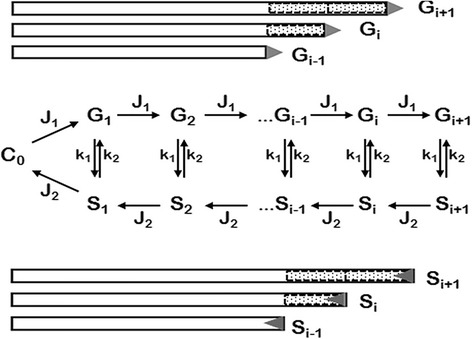
Kinetic model of MT growth adapted from Hill, 1984 [4]. G_i_ and S_i_ are the concentrations of growing and shrinking microtubules containing *i* number of tubulin dimers. C_0_ indicates MTs with zero length. J_1_ and J_2_ are the kinetic constants of growth and shrinking, respectively; k_1_, k_2_ are the constants for rates of growth-to-shrinking and shrinking-to-growth transitions. Growing and shrinking MTs are depicted as *bars*, and tubulin dimers are shown as *shaded bars*

One of the first theoretical description of microtubule growth and degradation [4] was based on data on microtubule dynamics obtained from in vitro experiments [5, 6]. Several kinetic models describing microtubule (MT) growth were proposed [4], and one of them, usually referred as the Hill’s model [4], is still widely used. According to this model, MT growth begins with the recruitment of tubulin dimers (present at the concentration *C*_*0*_) at a centrosome-associated nucleation site, followed by the polymerization of additional dimers at a rate constant *J*_*1*_ (growth phase) or depolymerization at a rate constant *J*_*2*_ (shrinking phase). At any length, the MTs may switch from the growth mode to a shrinking mode with a constant rate *k*_*1*_, or from a shrinking mode to a growth mode with a constant rate *k*_*2*_. A graphical representation of the Hill’s model is reported in Fig. 1. This kinetic model was later adapted to describe the polymerization/depolymerization kinetics of microtubules in *Xenopus* egg extracts and to analyze how cyclin A and cyclin B could affect this process [7]. In one of the kinetic regimes (bounded state), the experimental data were nicely fitting the model, and all of the constants could be defined. In addition to the bounded state where MTs are on average disassembling and *J*_*1*_ *k*_*2*_ *- J*_*2*_ *k*_*1*_ is negative, the authors also described an unbounded state (with *J*_*1*_ *k- J*_*2*_ *k' > 0*) where MTs are on average growing (our *k*_*1*_ and *k*_*2*_ are *k’* and *k* of *Hill* [4], respectively) [7].

The Hill’s model (Fig. 1) was applied to FRAP-based studies on pre-anaphase B/metaphase spindles of *Drosophila* syncytial embryos expressing GFP-tubulin [8]. The authors concluded that both bounded and unbounded regimes are inadequate to describe the observed FRAP dynamics. Data analysis indicated that tubulin dimers turn over almost entirely during a single cycle of MT shortening and growth, and consequently the recovery time does not depend on the size or position of the bleached region along the metaphase spindle. The recovery halftime (T_1/2_) after photobleaching is then simply the halftime of this cycle (i.e., *1/k*_*1*_ or *1/k*_*2*_ in terms of the model depicted in Fig. 1). This critical analysis served as a starting point for us to find other solutions to the kinetic scheme shown in Fig. 1.

Numerical modeling of a FRAP experiment [8] shows that the parameters that describe MT turnover can be determined from the modeling data. However, this approach is based on the mechanical model of Brust-Mascher et al. [9] and requires equations to be solved for the entire spindle, necessitating the knowledge of multiple mechanical parameters. As mentioned above, another approach to analyze MT dynamics is based on chemical kinetics description [4]. This approach operates with a smaller number of dynamic parameters and was verified by in vitro microtubule growth experiments. One of the objectives of the present study is developing a chemical-kinetic description for FRAP experiments on metaphase spindles. Another objective is the analysis of the dependence of spindle FRAP on parameters describing microtubule end dynamics. FRAP in a steady-state spindle has a simple relationship with the number of MT growing and shrinking ends within the photobleached area. The dynamics of these ends both with and without specific marks are not easy to evaluate, but can be inferred from the solution of system (2) for elementary intervals. Thus, our aim is describing the limit transition from the discrete Hill’s equations [4] to a continuous equation, and understanding how the kinetic constants of the Hill equation compare to those in the continuous equations. We found a solution for the Cauchy problem for partial differential equation (PDE) (2) for elementary intervals and used this expression to solve the equation for fluorescence recovery in a steady state spindle. Finally, we applied our model to the experimental data [8] and performed a quantitative analysis of FRAP recovery time.

## Results

### Limit passage to the continuous model

Since we were not able to solve discrete equations describing MT behavior with time, we are using a continuous model. We denote the MT concentration found in the growth phase as *G*_*i*_ *(t)*, where *i* is the number of polymerized tubulin dimers. Similarly, the concentration of microtubules in the shrinking phase is denoted as *S*_*i*_ *(t)*. Then, the kinetic equations for the concentration of tubulin dimer appear as:

1 $$ \left\{\begin{array}{c}\hfill \frac{\partial }{\partial t}{G}_i(t) = -{J}_1\left({G}_i(t)-{G}_{i-1}(t)\right) - {k}_1{G}_i(t)+{k}_2{S}_i(t)\hfill \\ {}\hfill \frac{\partial }{\partial t}{S}_i(t) = -{J}_2\left({S}_i(t)-{S}_{i+1}(t)\right) - {k}_2{S}_i(t)+{k}_1{G}_i(t)\hfill \end{array}\right. $$

We can rewrite equation (1) as follows, considering *Δx* is a small coordinate step:

$$ \left\{\begin{array}{c}\hfill \frac{\partial }{\partial t}{G}_i(t) = -{J}_1\frac{\varDelta x}{\varDelta x}\left({G}_i(t)-{G}_{i-1}(t)\right) - {k}_1{G}_i(t)+{k}_2{S}_i(t)\hfill \\ {}\hfill \frac{\partial }{\partial t}{S}_i(t) = -{J}_2\frac{\varDelta x}{\varDelta x}\left({S}_i(t)-{S}_{i+1}(t)\right) - {k}_2{S}_i(t)+{k}_1{G}_i(t)\hfill \end{array}\right. $$

The values *v*_*1*_ *= J*_*1*_ *Δx* and *v*_*2*_ *= J*_*2*_ *Δx* have a dimension of *cm/sec*, which correspond to the linear polymerization and depolymerization speeds of a single tubulin dimer. If *Δx* is negligible compared to the microtubule size, the above equations can be transformed into PDEs, where *t* is a time and *x* is a coordinate:

2 $$ \left\{\begin{array}{c}\hfill \frac{\partial }{\partial t}G\left(x,t\right) = -{v}_1\left[\frac{\partial }{\partial x}G\left(x,t\right)\right] - {k}_1G\left(x,t\right)+{k}_2S\left(x,t\right)\hfill \\ {}\hfill \frac{\partial }{\partial t}S\left(x,t\right) = {v}_2\left[\frac{\partial }{\partial x}S\left(x,t\right)\right] - {k}_2S\left(x,t\right)+{k}_1G\left(x,t\right)\hfill \end{array}\right. $$

As reported below, this transformation will help us to find a mathematical solution for MT behavior.

### Stationary regime and bounded state

Let us solve the equations for the stationary regime, i.e., when the process does not depend on time, namely when *t = 0*. In this case, *G(x)* and *S(x)* denote the distribution of growing and shrinking ends over the coordinate *x*, which corresponds to the current length of MTs.

$$ \left\{\begin{array}{c}\hfill {v}_1\left[\frac{\partial }{\partial x}G(x)\right]= - {k}_1G(x)+{k}_2S(x)\hfill \\ {}\hfill\ {v}_2\left[\frac{\partial }{\partial x}S(x)\right]= - {k}_1G(x)+{k}_2S(x)\hfill \end{array}\right. $$

The discriminant of this system,

$$ D={\left(\frac{k_1}{v_1}-\frac{k_2}{v_2}\right)}^2 $$

is either positive or zero. Then a characteristic equation:

$$ {\lambda}^2-\left(\frac{k_2}{v_2}-\frac{k_1}{v_1}\right)\lambda =0 $$

has two roots,

$$ {\lambda}_1=0,\kern1em {\lambda}_2 = \frac{k_2{v}_1-{k}_1{v}_2}{v_1{v}_2} = -\alpha $$

Substituting the experimental values of *k*_*1*_ *; k*_*2*_ *; v*_*1*_ *; v*_*2*_ from reference [10] into the expression for *λ*_*2*_ results in a positive *α*. The general solution of the system (3) is

$$ \left\{\begin{array}{c}\hfill G(x) = {C}_1\frac{k_2}{v_1}+{C}_2\frac{k_2}{v_1}{e}^{-\alpha x}\ \hfill \\ {}\hfill S(x) = {C}_1\frac{k_1}{v_1}+{C}_2\frac{k_2}{v_2}{e}^{-\alpha x}\hfill \end{array}\right. $$

where *C*_*1*_ and *C*_*2*_ are arbitrary constants. If at *x = 0; G(x) = G*_*0*_ *,* and upon *x* tending to infinity *G(x) = G*_*∞*_, then

$$ \left\{\begin{array}{c}\hfill G(x) = {G}_{\infty } + \left({G}_0 - {G}_{\infty}\right){e}^{-\alpha x}\ \hfill \\ {}\hfill S(x) = \frac{k_1}{k_2}{G}_{\infty } + \left({G}_0 - {G}_{\infty}\right)\frac{v_1}{v_2}{e}^{-\alpha x}\hfill \end{array}\right. $$

**Fig. 2:**
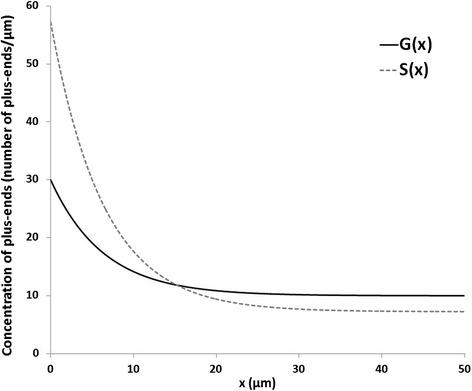
Concentrations of growing and shrinking microtubule ends as a function of coordinate x for a model case

Using the experimentally observed values of dynamic parameters [10] and arbitrarily assigning *G*_*0*_ *= 20* and *G*_*∞*_ *= 30*, the system can be visualized as shown in Fig. 2.

If *x* is measured in *μm*, the *Gk*_*1*_ *= Sk*_*2*_ ratio holds true for a wide range of large *x* values, whereas in the case of small *x* values, which is most pertinent to the experimental situation, an alternative ratio should be applied. A negative-going exponential function for microtubule length (at *G*_*∞*_ *= 0*) observed for the discrete form of equations (1) in the stationary regime (at *v*_*2*_ *k*_*1*_ *> v*_*1*_ *k*_*2*_) was discussed earlier [7] and was successfully used to interpret the MT length distribution in experiments in vitro. Based on these experimental data [7] and given the finite number of tubulin molecules in the cell, *G*_*∞*_ *> 0* is not a viable option and so *G*_*∞*_ should be set as *0*. In this case, a simple ratio *G(x)v*_*1*_ *= S(x)v*_*2*_ is true and corresponds to a situation where the spindle remains unchanged with time, and the numbers of growing and shrinking microtubules are equal.

### Analytical solution of the equations for elementary intervals

The system of continuous equations (2) was first published by *Dogterom and Leibler* [11]; the authors stated that it could be solved analytically with the border conditions set at *t = 0*, and with all MTs having zero length. However, they did not publish the solution. In the following section we find this solution.

The solution refers to a FRAP experiment that involves photobleaching of fluorescently labeled MTs in a rectangular region of width *L* perpendicular to the spindle axis, followed by the analysis of the dynamics of fluorescence recovery in the bleached region. In a real experiment both *G* (Growing) and *S* (Shrinking) MT ends are distributed uniformly across this region at the initial time-point after bleaching. Let us consider the elementary interval *Δ* of *L*, which initially contains *G*_*0*_ and *S*_*0*_ unmarked ends (in the stationary case *G*_*0*_ *v*_*1*_ *= S*_*0*_ *v*_*2*_ *; v*_*1*_ and *v*_*2*_ are the velocities of growth and shrinking, respectively). The *G*_*0*_ ends will start to produce a marked tubulin track and will move towards the spindle equator from the elementary interval, while the *S*_*0*_ ends will move to the opposite direction. Some *G*_*0*_ ends will then turn into *S* ends (catastrophe) and some *S*_*0*_ ends will turn into *G* ends (rescue). To describe the behavior of *G*_*0*_ and *S*_*0*_ ends it is necessary to solve the Cauchy problem for (2). We thus substituted the *x* and *t* variables with *α* and *β*, and converted the functions *G(x; t)* and *S(x; t)* into *u(α;β)* and *v(α;β)*:

$$ \left\{\begin{array}{c}\hfill \alpha =x - {v}_1t\hfill \\ {}\hfill \beta =x + {v}_2t\hfill \end{array}\right.\kern1em \left\{\begin{array}{c}\hfill G\left(x,t\right)=u\left(\alpha, \beta \right){e}^{-\alpha \beta + ba}\hfill \\ {}\hfill S\left(x,t\right)=v\left(\alpha, \beta \right){e}^{-\alpha \beta + ba}\hfill \end{array}\right.\kern1.25em \left\{\begin{array}{c}\hfill a=\frac{k_1}{v_1+{v}_2}\hfill \\ {}\hfill b=\frac{k_2}{v_1+{v}_2}\hfill \end{array}\right. $$

We obtain the equation system for the functions *u(α; β)* and *v(α; β)*:

$$ \begin{array}{c}\hfill \frac{\partial }{\partial \beta }u\left(\alpha, \beta \right)=bv\left(\alpha, \beta \right)\hfill \\ {}\hfill \frac{\partial }{\partial \alpha }v\left(\alpha, \beta \right)=bv\left(\alpha, \beta \right)\hfill \end{array} $$

The elimination of *v(α; β)* gives us the following equation for *u(α; β)*:

3 $$ \frac{\partial }{\partial \beta}\left(\frac{\partial }{\partial \beta }u\left(\alpha, \beta \right)\right)+a\ b\ u\left(\alpha, \beta \right)=0 $$

With *u(α; β)* known, one can determine *v(α; β)* from the first equation of the system. If *u(α;β)*_*α=β*_ *= u*_*0*_ *(x)* and *v(α;β)*_*α=β*_ *= v*_*0*_ *(x)* are known, we can supply equation (3) with the boundary condition:

$$ \begin{array}{c}\hfill u\left(\alpha, \beta \right)\Big|{}_{\alpha =\beta }={u}_0(x)\hfill \\ {}\hfill \frac{\partial }{\partial \beta }u\left(\alpha, \beta \right)\Big|{}_{\alpha =\beta }=b{v}_0(x)\hfill \end{array} $$

The solution of this equation, already presented in ref [12], is expressed as:

4 $$ u\left(\alpha, \beta \right) = {u}_0\left(\alpha \right)+b{\displaystyle \underset{\alpha }{\overset{\beta }{\int }}}{I}_0\left[2\sqrt{ab}\sqrt{\left(\xi -\alpha \right)\left(\beta -\xi \right)}\right]{v}_0\left(\xi \right)d\xi +\sqrt{ab}{\displaystyle \underset{\alpha }{\overset{\beta }{\int }}}{I}_1\left[2\sqrt{ab}\sqrt{\left(\xi -\alpha \right)\left(\beta -\xi \right)}\right]\sqrt{\frac{\beta -\xi }{\xi -\alpha }}{u}_0\left(\xi \right)d\xi $$

By reverting to variables *x* and *t* and functions *G(x; t)* and *S(x; t)* and by denoting their values at *t = 0* as *G*_*0*_ *(x)* and *S*_*0*_ *(x)*, we obtain:

$$ G\left(x,t\right) = = {G}_0\left(x-{v}_1t\right){e}^{-{k}_1t} + \frac{k_2}{v_1+{v}_2}{e}^{\frac{-\left({k}_1{v}_2+{k}_2{v}_1\right)}{v_1+{v}_2}t}{\displaystyle {\int}_{x-{v}_1t}^{x+{v}_2t}{I}_0}\left(2\frac{\sqrt{k_1{k}_2}\sqrt{\left(\xi -x+{v}_1t\right)\left(x+{v}_2t-\xi \right)}}{v_1+{v}_2}\right){S}_0\left(\xi \right){e}^{\frac{\left({k}_2-{k}_1\right)\left(x-\xi \right)}{v_1+{v}_2}}d\xi +\frac{\sqrt{k_1{k}_2}}{v_1+{v}_2}{e}^{\frac{-\left({k}_1{v}_2+{k}_2{v}_1\right)}{v_1+{v}_2}t}{\displaystyle {\int}_{x-{v}_1t}^{x+{v}_2t}{I}_1}\left(2\frac{\sqrt{k_1{k}_2}\sqrt{\left(\xi -x+{v}_1t\right)\left(x+{v}_2t-\xi \right)}}{v_1+{v}_2}\right){G}_0\left(\xi \right)\cdot \cdot {e}^{\frac{\left({k}_2-{k}_1\right)\left(x-\xi \right)}{v_1+{v}_2}}\sqrt{\frac{\left(x+{v}_2t-\xi \right)}{\left(\xi -x+{v}_1t\right)}}d\xi $$

5 $$ S\left(x,t\right)=={S}_0\left(x+{v}_2t\right){e}^{-{k}_2t}+\frac{k_1}{v_1+{v}_2}{e}^{\frac{-\left({k}_1{v}_2+{k}_2{v}_1\right)}{v_1+{v}_2}t}{\displaystyle {\int}_{x-{v}_1t}^{\kern0.28em x+{v}_2t}{I}_0}\left(2\frac{\sqrt{k_1{k}_2}\sqrt{\left(\xi -x+{v}_1t\right)\left(x+{v}_2t-\xi \right)}}{v_1+{v}_2}\right){e}^{\frac{\left({k}_2-{k}_1\right)\left(x-\xi \right)}{v_1+{v}_2}}{G}_0\left(\xi \right)d\xi +\frac{\sqrt{k_1{k}_2}}{v_1+{v}_2}{e}^{\frac{-\left({k}_1{v}_2+{k}_2{v}_1\right)}{v_1+{v}_2}t}{\displaystyle {\int}_{x-{v}_1t}^{x+{v}_2t}{I}_1}\left(2\frac{\sqrt{k_1{k}_2}\sqrt{\left(\xi -x+{v}_1t\right)\left(x+{v}_2t-\xi \right)}}{v_1+{v}_2}\right)\cdot {e}^{\frac{\left({k}_2-{k}_1\right)\left(x-\xi \right)}{v_1+{v}_2}}\sqrt{\frac{\left(\xi -x+{v}_1t\right)}{\left(x+{v}_2t-\xi \right)}}{S}_0\left(\xi \right)d\xi $$

Next, we consider *G*_*0*_ *(x) = G*_*00*_ *Im(x)* and *S*_*0*_ *(x) = S*_*00*_ *Im(x) = (v*_*1*_ */v*_*2*_ *)G*_*00*_ *Im(x)* as short pulses along the *x* axis having the width *Δ*, which is small compared to the interval *L*, where

$$ Im(x) = \left\{\begin{array}{c}\hfill 1,\kern0.5em  if\ 0<x<\varDelta; \hfill \\ {}\hfill 0,\kern0.5em  otherwise\kern1em ,\hfill \end{array}\right. $$

*G*_*00*_ is a linear concentration of *G* ends at the initial time-point. Numerical calculations based on various values of *v*_*1*_ *; v*_*2*_ *; k*_*1*_ *; k*_*2*_ show that the formulae (5) indeed represent the solutions of equation (2). It must be noted that the pair of functions:

$$ \left\{\begin{array}{c}\hfill G\left(x,t\right) = {e}^{\frac{k_2\left(x+{v}_2t\right)}{v_1+{v}_2} - \frac{k_1\left(x-{v}_1t\right)}{v_1+{v}_2}}\hfill \\ {}\hfill S\left(x,t\right) = {e}^{\frac{k_2\left(x+{v}_2t\right)}{v_1+{v}_2} - \frac{k_1\left(x-{v}_1t\right)}{v_1+{v}_2}}\hfill \end{array}\right. $$

also represent a particular solution of equation (2). By using this solution at *t = 0* in (4), we confirmed that the downstream substitution of the expressions obtained in (2) results in equality. Since the expression (5) cannot be easily processed numerically, we approximated the functions *G(x; t)* and *S(x; t)* by the simpler formulae *GF(x; t)* and *SF(x; t)*. These formulae represent an expansion of expression (5), as follows:

6 $$ \begin{array}{l}GF\left(x,t\right)= = \left\{\begin{array}{c}\hfill {e}^{\frac{k_2-{k}_1}{v_1+{v}_2}x+\frac{-\left({k}_1{v}_2+{k}_2{v}_1\right)}{v_1+{v}_2}t}{G}_{00}\varDelta \left[\frac{k_2{v}_1}{\left({v}_1+{v}_2\right){v}_2}{I}_0\left[2\frac{\sqrt{k_1{k}_2}\sqrt{\left(-x+{v}_1t\right)\left(x+{v}_2t\right)}}{v_1+{v}_2}\right]+\frac{\sqrt{k_1{k}_2}}{\left({v}_1+{v}_2\right)}{I}_1\left[2\frac{\sqrt{k_1{k}_2}\sqrt{\left(-x+{v}_1t\right)\left(x+{v}_2t\right)}}{v_1+{v}_2}\right]\sqrt{\frac{\left(x+{v}_2t\right)}{\left(-x+{v}_1t\right)}}\right],\kern0.24em  if-{v}_2t+\varDelta <x<{v}_1t\hfill \\ {}\hfill {G}_{00}Im\left(x-{v}_1t\right){e}^{-{k}_1t},\kern0.24em  if\;{v}_1t\le x\le {v}_1t+\varDelta \hfill \\ {}\hfill 0,\kern0.75em  otherwise\hfill \end{array}\right.\ \\ {}SF\left(x,t\right)= = \left\{\begin{array}{c}\hfill {e}^{\frac{k_2-{k}_1}{v_1+{v}_2}x+\frac{-\left({k}_1{v}_2+{k}_2{v}_1\right)}{v_1+{v}_2}t}{G}_{00}\varDelta \left[\frac{k_1}{\left({v}_1+{v}_2\right)}{I}_0\left[2\frac{\sqrt{k_1{k}_2}\sqrt{\left(-x+{v}_1t\right)\left(x+{v}_2t\right)}}{v_1+{v}_2}\right]+\frac{\sqrt{k_1{k}_2}}{\left({v}_1+{v}_2\right)}\frac{v_1}{v_2}{I}_1\left[2\frac{\sqrt{k_1{k}_2}\sqrt{\left(-x+{v}_1t\right)\left(x+{v}_2t\right)}}{v_1+{v}_2}\right]\sqrt{\frac{\left(-x+{v}_1t\right)}{\left(x+{v}_2t\right)}}\right],\kern0.24em  if-{v}_2t+\varDelta <x<{v}_1t\hfill \\ {}\hfill {G}_{00}\frac{v_1}{v_2}Im\left(x+{v}_2t\right){e}^{-{k}_2t},\kern0.5em  if - {v}_2t\le x\le -{v}_2t+\varDelta \hfill \\ {}\hfill 0,\kern0.75em  otherwise\hfill \end{array}\right.\end{array} $$

**Fig. 3:**
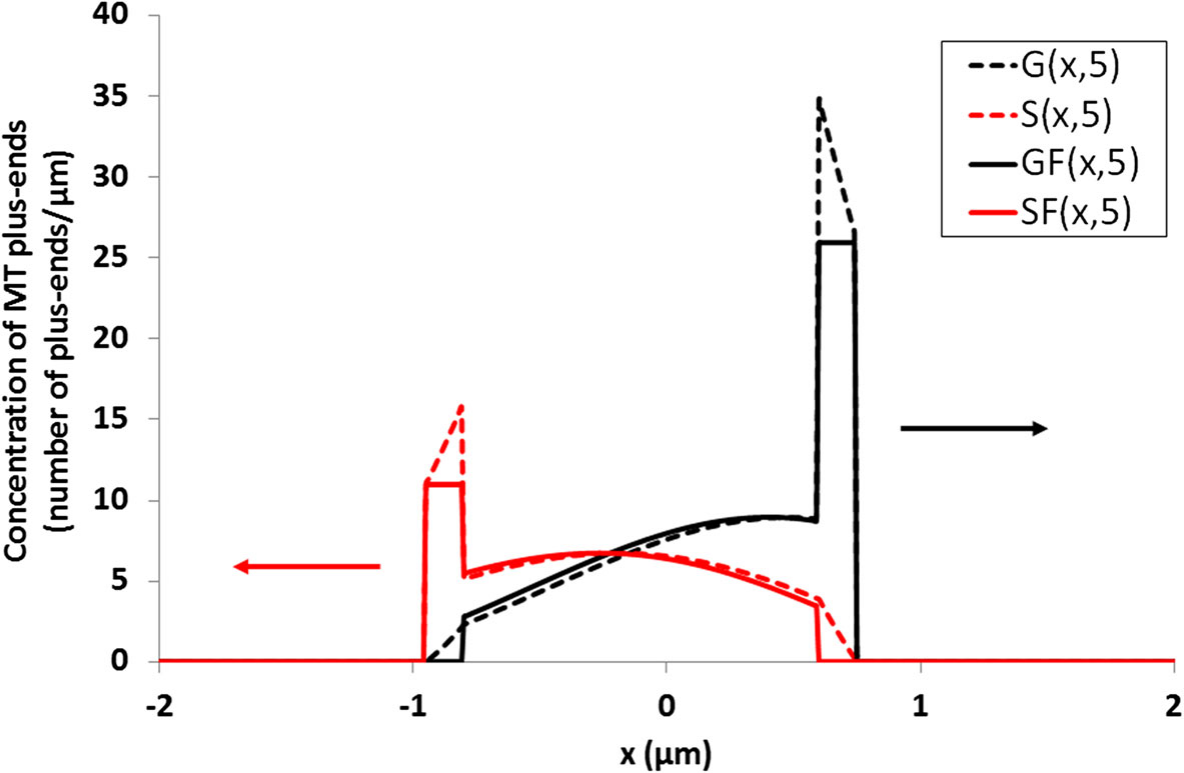
The behavior of the solution of (5) and the approximate solution of (6) at t = 5 are depicted as *dashed* and *continuous lines*, respectively; *black* and *red* limes refer to growing and shrinking MTs, respectively. The parameters used are v_1_ = 0.12 μm/s, v_2_ = 0.19 μm/s, k_1_ = 0.27 1/s, k_2_ = 0.35 1/s, G_00_ = 100. The length of the photobleached area is L = 3 μm, and the short pulse width Δ = 0.15 μm. *Arrows* denote the direction of the G(x; t) and S(x; t) front movements

We are now trying to determine the evolution of short pulses initially situated within a small Δ interval. A short pulse is concentration of growing (G) or shrinking (S) ends of x length in a small Δ interval at a given time. The analysis for short pulses located close to the origin according to equation (2) is shown in Fig. 3. The short pulses become exponentially weaker with time: the short pulse *G* moves right at a velocity *v*_*1*_ and its height decreases with time at a rate constant *k*_*1*_. The short pulse *S* moves left at a velocity *v*_*2*_, and its height decreases exponentially with time at a rate constant *k*_*2*_. We note that the asymptotic approximation we used matches the exact solution fairly well. It only differs from the exact solution at the top of the short pulse. However, taking into account that the short pulse width *Δ* is small, this does not result in a significant distortion of the solution.

In conclusion, we found the solution of the system of continuous equations (2). An important contribution of this solution is the possibility to differentiate between labeled and unlabeled *S* ends. As can be seen in Fig. 3, the *S* ends on the left of the *x = 0* coordinate point are unlabeled, whereas those on the right are labeled.

### Solution for finite x coordinate intervals

As mentioned, we found a solution for (2) for evolution of concentration "pulse" *Δ* of G and S ends initially located at *x = 0*. In this case, after time *t*, *G* and *S* ends at the point *r* located to the right of *x = 0* gave a marked tubulin track with length *r*, while the ends on the left did not leave marked tracks. Thus, a positive *r* can be considered as the length of a marked track. In this section we are constructing the solution where *G* and *S* ends are initially distributed over the *0-L* interval of *x* coordinate (in a real photobleaching experiment *Δ* could not be negligible compared to the spindle length) and are then moving in different directions according to (2). This can be done if the solution (6) is convolved with a rectangle over *x* (*Rect(x) = 1* if *0 < x < L* and *Rect(x) = 0* otherwise). Since for the following analysis we need only the degrading *S* ends that have no tracks, the auxiliary integration variable *xx* must be negative and vary from *-L* to *0*. However, when integrating over the initial position of the impulse at *0-Δ*, we need to move the limits of *xx* to *-L + Δ*, and consider that the *Δ* interval introduces a small error (*Δ* is small). The concentration *S*_*tail*_ *(x,t)* of *S* ends without tracks (integrated over initial position of the pulse) at the coordinate point *x* and at the time *t* is:

$$ {S}_{tail}\left(x,t\right) = \frac{1}{L}{\displaystyle \underset{-L+\Delta}{\overset{\varDelta }{\int }}} Rect(x)\;SF\left(xx+x,t\right)\;dxx $$

The total number of degrading *S* ends without a tubulin label could be determined by the integration of *S*_*tail*_ *(x,t)* over *x*. With *t = 0*, the total number of *S* ends is *G*_*00*_ *Δv*_*1*_ */v*_*2*_ (at the stationary spindle *G*_*00*_ *v*_*1*_ *= S*_*00*_ *v*_*2*_, see above); therefore, the fraction *S*_*n*_ *(t)* of *S* ends (without a label) among all *S* ends is:

$$ {S}_n(t)=\frac{v_2}{v_1{G}_{00}\varDelta }\ {\displaystyle \underset{0}{\overset{L}{\int }}}{S}_{tail}\left(x,t\right)dx $$

This expression permits construction of a solution for a stationary spindle.

### FRAP in a stationary spindle

The essential changes in the mitotic spindle structure occur during prometaphase and at the metaphase-to-anaphase transition. During metaphase the spindle shape is relatively stable, so that the stationary state appears a good approximation.

In FRAP experiments on mitotic spindles containing fluorescent tubulin, a rectangular area perpendicular to the spindle axis is photobleached, and fluorescence recovery in this area is recorded [8]. Let us consider a stationary case (see above) where:

$$ \begin{array}{c}\hfill G(x) = {G}_0\ {e}^{-\alpha x}\hfill \\ {}\hfill S(x) = {G}_0\ \frac{v_1}{v_2}{e}^{-\alpha x}\hfill \end{array} $$

In this case, the concentration of *G* ends in the photobleached area is constant with all *G* ends incorporating marked tubulin dimers. At any given time, the concentration of *S* ends is also constant, but *S* ends can be of two types: *S*_*n*_ ends that degrade releasing of non-marked photobleached tubulin, and *S*_*m*_ ends that shrink after a short growth phase and therefore degrade releasing fluorescent tubulin.

The fluorescence recovery detection area is usually small compared to the spindle length, and we can neglect concentration changes within this zone. Therefore, we can use *G*_*0*_ and *S*_*0*_ *= G*_*0*_ *v*_*1*_ */v*_*2*_ instead of *G(x)* and *S(x)*; because the spindle is stationary, *G*_*0*_ and *S*_*0*_ do not depend on time. If large intervals are considered, the equation would need only to account for the exponential dependence of *G(x)* and *S(x)* over coordinate *x*.

Growing *G*_*0*_ ends incorporate marked tubulin at a speed of *v*_*1*_, while the fraction of degrading *S*_*m*_ *(t)* ends release marked tubulin at a speed *v*_*2*_. If *M(t)* is the length of marked MTs within the bleached area, its time derivative would be:

$$ \frac{d}{dt}M(t) = {v}_1{G}_0 - {v}_2{S}_m(t) $$

By introducing the fraction of marked tubulin *Lb(t) = M(t)/E*, where *E* is the total length of marked MTs within the zone before photobleaching, and by accounting for *G*_*0*_ *v*_*1*_ *= S*_*0*_ *v*_*2*_ and *S*_*m*_ *(t) = S*_*0*_ *(1-S*_*n*_ *(t))*, we obtain:

$$ \frac{d}{dt}Lb(t) = \frac{v_2{S}_0}{E}{S}_n(t) $$

Integration leads to:

$$ Lb(t)-Lb\left({t}_0\right) = \frac{v_2{S}_0}{E}{\displaystyle \underset{t_0}{\overset{t}{\int }}}{S}_n(t)dt $$

Before photobleaching, most *G* and *S* ends within the *L* interval have marked tubulin at their ends and are related by *G*_*0*_ *v*_*1*_ *= S*_*m*_ *v*_*2*_. After fluorescence recovery (when the *S*_*n*_ ends have disappeared), the *G*_*0*_ *v*_*1*_ *= S*_*m*_ *v*_*2*_ condition is again present. In such cases, the track length for both *G*_*0*_ and *S*_*m*_ ends before and after fluorescence recovery would be *L/2*. Fluorescence recovery normalization to the level of fluorescence before bleaching is the accepted mode of experimental data analysis. In our model, the fluorescence levels before bleaching and after full recovery coincide. Thus, we can normalize the theoretical dependence fluorescence level after full recovery:

7 $$ \frac{Lb(t)-Lb\left({t}_0\right)}{Lb\left(\infty \right)-Lb\left({t}_0\right)} = \frac{{\displaystyle {\int}_{t_0}^t}{S}_n(t)dt}{{\displaystyle {\int}_{t_0}^{\infty }}{S}_n(t)dt} $$

(7) is the final dependence that can be used to fit the experimental spindle FRAP curves. It is clear that (7) does not depend on *v*_*2*_ *S*_*0*_ */E*.

### Numerical implementation

**Fig. 4:**
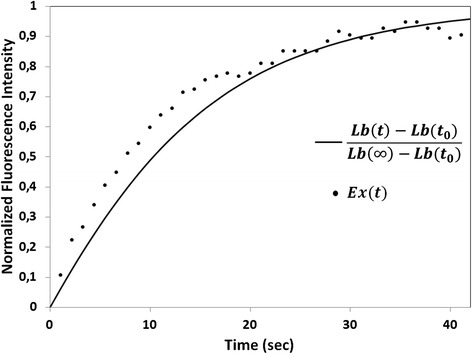
Experimental curve for pre-anaphase B [8] and its theoretical approximation (reproduced with permission of the authors [8]). Ex(t) are the corrected experimental points from [8], i.e., the fluorescence level detected just after photobleaching was subtracted from the experimental values; experimental points were then normalized to the fluorescence level before photobleaching. The theoretical photobleaching curve (*continuous line*) was calculated for various values of v_1_, v_2_, k_1_, and k_2_ using Mathcad-14 software

A pre-anaphase-B FRAP curve (near the equator) for *Drosophila* syncytial embryo mitosis was obtained by *Cheerambathur* et al. [8], who also proposed a theoretical model for the FRAP curve and determined the dynamic parameters of the *G* and *S* ends. Approximate parameter values are *v*_*1*_ *= v*_*2*_ *= 0.35 μm/s, k*_*1*_ *= 0.2 1/s* and *k*_*2*_ *= 0.25 1/s*. The width of the photobleached area near the equator was *2.2 μm*. They [8] also reported the speed of EB1 labeled ends (*0.25 μm/s*) in their experimental system. In our model, like in the theoretical model of [8]), *v*_*1*_ and *v*_*2*_ are the speeds in the photobleached area, which is slowly moving to the pole at the speed of the flux (*0.05 μm/s* according to [8]). Thus, we calculated *0.25-0.05 μm/s* for *v*_*1*_, considered *k*_*1*_ *= 0.2 1/s,* and performed minimization of the root-mean-square deviation of our theoretical curve (7) from the experimental one [8]. The simple gradient descent method was used to find a local minimum near the dynamic parameters of [8]. The theoretical and experimental curves are shown in Fig. 4; the optimal parameters are *v*_*1*_ *= 0.2 μm/s, v*_*2*_ *= 0.08 μm/s, k*_*1*_ *= 0.2 1/s,* and *k*_*2*_ *= 0.4 1/s*.

In our model, we assume that full fluorescence recovery occurs within the bleached area, regardless its position along the spindle. However, there is experimental evidence that fluorescence recovery is higher near the spindle equator than at the poles [8, 13, 14]. It should be also mentioned that in the stationary case, *G* and *S* ends are distributed over the half-spindle in an exponential manner (with exponent index *–αx*), with the maximum near the pole and the minimum near the equator. Thus, photobleaching near the poles will not only affect MT ends situated in the bleached area but also the ends of “MT fragments” detached from the poles; fluorescence of these “MT fragments” will not be restored during the experiment, lowering the overall level of fluorescence recovery. If the bleached area is near the equator, this effect would be small.

## Discussion

The FRAP technique, although widely used for studying the dynamics of spindle MT behavior, has still some limitations. These limitations are due to the fact that the kinetic constant value measured in the experiments (referred to as the "rate of synthetic processes in the spindle") has no direct link to the dynamic parameters of MTs. The work of *Cheerambathur* et al. [8] partially addressed this issue by linking this parameter with *k*_*1*_ and *k*_*2*_, but the authors have not solved the problem completely. This is because modeling of the fluorescence recovery curve proposed earlier by *Brust-Mascher* et al. [9] requires the mechanical equations for the entire spindle to be solved, which in turn requires the knowledge of multiple mechanical parameters. Here, we provided an alternative model for the interpretation of the fluorescence recovery curve. Previously published reports describing MT polymerization and depolymerization dynamics have served as the starting point for our analysis. Based on these studies, we analytically solved the PDE, which describes the dynamics and transitions between the states of MT ends. We then used this solution to solve the equation describing fluorescence recovery in a steady-state spindle and, finally, we obtained a theoretical dependence for the fluorescence recovery curve. Our study provides a model for the FRAP recovery curve if all four parameters are known. We also made a model calculation to determine how lowering of each parameter could affect *T*_*1/2*_ FRAP time. As shown in Table 1, lowering of *v*_*1*_ or *k*_*2*_ leads to an increase in *T*_*1/2*_, while decrease of *v*_*2*_ or *k*_*1*_ decreases *T*_*1/2*_.

**Table 1:** Alterations of MT growth/shrinking parameters affect T_1/2_ FRAP times for half spindles (calculations according to our model)

v_1_ (μm/s)	v_2_ (μm/s)	k_1_ (1/s)	k_2_ (1/s)	T_1/2_ (sec)
0.25	0.09	0.21	0.17	**31**
0.24	0.09	0.21	0.17	**33**
0.25	0.09	0.21	0.17	25
0.25	0.09	0.20	0.17	28
0.25	0.09	0.21	0.16	**36**

The lower parameter is underlined. Increased and decreased FRAP times are indicated by bold large numbers and small numbers, respectively

Some mutant proteins decrease the rate of the process in which they are involved compared to their wild type counterparts, so we made an attempt to analyze published FRAP data for three mutant *Drosophila* mitotic proteins. Mini-spindles (Msps), a member of the XMAP215/TOG protein family, concentrates not only at the centrosomes but also at the MT plus-end [14]. Based on their own data and pre-existing data *Buster* et al. [14] concluded that Msps positively regulates transition from pausing to MT growth state. Although our model does not include MT pausing, a decreased transition to growth means that the constant *k*_*2*_ is decreased. Table 1 shows that the decrease in *k*_*2*_ results in an increase in the FRAP time, which is what has been in fact observed by *Buster* et al. [14]. A second example is concerned with the Mast/Orbit protein, a CLASP family protein that is found at microtubule plus-ends near the kinetochore [15, 16]. RNAi-mediated depletion of Mast/Orbit leads to MT flux inhibition [14], consistent with the finding that Mast is involved in the control of microtubule polymerization [17]. Thus, Mast affects *v*_*1*_ speed in our model and a mutation in *Mast* would decrease *v*_*1*_. The *v*_*1*_ decrease (Table 1) decreases FRAP time, according to the observations of *Buster* et al. [14]. In a third example we consider the Eb1 protein, a well-known microtubule plus-end marker that increases MT rescue frequency while decreasing pause [14]. Loss of Eb1 would therefore decrease the *k*_*2*_ parameter, which would lead to an increase in FRAP time as has been in fact observed [14].

The empirical FRAP curve of *Salmon* et al. [3] permits determination of the dynamic parameter commonly called "tubulin turnover". Here, we have shown that the spindle FRAP curve depends on four kinetic parameters of MT end polymerization and depolymerization. Using the results of *Cheerambathur* et al. [8], we showed that the solution we found can adequately approximate our experimental FRAP curve for metaphase spindles. In addition, we posited that differences in fluorescence recovery between the poles and the equator of metaphase spindles, which are commonly observed in FRAP experiments, could be explained by the exponential distribution of *G* and *S* ends in the half-spindle as proposed in our model.

## Conclusions

Here, we provided an alternative model for the interpretation of the fluorescence recovery curve. Based on the previously published reports describing MT polymerization and depolymerization dynamics, we analytically solved the PDE, which describes the dynamics and transitions between the states of MT ends. We then used this solution to solve the equation describing fluorescence recovery in a steady-state spindle and, finally, we obtained a theoretical dependence for the fluorescence recovery curve. A numerical expression that describes the distribution of polymerizing and depolymerizing microtubule ends as a function of four kinetic parameters is presented. We also made a model calculation to determine how lowering of each parameter could affect *T*_*1/2*_ FRAP time. As shown in this study, the lowering of *v*_*1*_ or *k*_*2*_ leads to an increase in *T*_*1/2*_, while the decrease of *v*_*2*_ or *k*_*1*_ decreases *T*_*1/2*_. The numerical expression we elaborated can replace the empirical FRAP curve described earlier for a spindle comprising fluorescently marked microtubules. The curve we generated fits well the experimental data.

## Abbreviations

FRAP: Fluorescence recovery after photobleaching
GFP: Green fluorescent protein
MT: Microtubule
PDE: Partial differential equation
